# Features of event-related potentials during retrieval of episodic memory in patients with mild cognitive impairment due to Alzheimer’s disease

**DOI:** 10.3389/fnins.2023.1185228

**Published:** 2023-07-04

**Authors:** Yingying Wang, Xing Ye, Bo Song, Yixin Yan, Wenyin Ma, Jingping Shi

**Affiliations:** Department of Neurology, Affiliated Nanjing Brain Hospital, Nanjing Medical University, Nanjing, China

**Keywords:** event-related potential, electroencephalography, mild cognitive impairment, Alzheimer’s disease, recognition memory

## Abstract

**Objective:**

To provide a rigorous comparison between patients with mild cognitive impairment due to Alzheimer’s disease (MCI-AD) and healthy elderly, as well as to assess the value of electroencephalography (EEG) in terms of early diagnosis, we conducted a neutral image recognition memory task involving individuals with positive biomarkers including β amyloid deposition, pathologic tau or neurodegeneration.

**Methods:**

The task involving study and test blocks was designed to evaluate participants’ recognition memory. Electroencephalogram was recorded synchronously to elicit event-related potentials in patients with MCI-AD and healthy control subjects. We further analyzed differences between groups or conditions in terms of behavioral performance, time domain, and time-frequency domain.

**Results:**

The MCI-AD cohort showed a slower response time to old/new images and had low accuracy regarding behavioral performance. The amplitude of the late positive complex for the old/new effects was significantly suppressed in the MCI-AD cohort when compared with that in the HC cohort. The amplitude of the late old/new effects was correlated with the Auditory Verbal Learning Test recognition score in all participants. The time-frequency domain analysis revealed that correct recognition of old items elicited a decrease in beta power, mainly limited to the HC cohort. Moreover, the combination of behavioral (processing speed and accuracy) and electrophysiological (average amplitude and relative power of delta band) measures contributes to classifying patients with MCI-AD from healthy elderly people.

**Conclusion:**

Changes of old/new effects, accuracy and response time are sensitive to the impairment of recognition memory in patients with MCI-AD and have moderate value in predicting the incipient stage of AD.

## 1. Introduction

According to the World Health Organization, more than 55 million people worldwide live with dementia, as reported in 2022, which has imposed a huge economic and healthcare burden (Jia et al., 2018). Alzheimer’s disease (AD) is the major type of dementia, accounting for 60–70% of the total dementia cases (Ferreira et al., 2014). In the absence of a cure, risk reduction, timely diagnosis, and early intervention have become the mainstay of people’s attention (Robinson et al., 2015). However, Alzheimer’s Disease International has reported that 75% of people who live with dementia worldwide are not diagnosed clinically and that is not to mention about post-diagnosis support (Bamford et al., 2021).

Mild cognitive impairment due to AD (MCI-AD) is the symptomatic predementia phase of AD, and such people may progress to AD-related dementia over time (Albert et al., 2011). However, people who receive effective interventions may have longer disability-free life expectancy. In AD, extracellular amyloid-β (Aβ) aggregates and intracellular tau neurofibrillary tangles are typical pathological features that can persist for more than two decades before the onset of symptoms (Villemagne et al., 2018). These pathological changes can damage synapse structure and function, thereby promoting neurodegeneration and resulting in cognitive impairment (Sheng et al., 2012). Although various techniques enable accurate in-vivo assessment of AD biology through cerebrospinal fluid (CSF) analysis or amyloid positron emission tomography imaging, their application is slightly restricted for individuals with preclinical AD or for large-scale screening considering invasiveness, time, and cost concerns. Thus, newer tools or technology is required to improve the convenience and accuracy of early diagnosis.

Tools such as quantitative electroencephalography (qEEG) or event-related potentials (ERPs) are suitable not only for studying neurocognitive processes because they directly measure neural activity and may provide sensitive biomarkers for the early diagnosis of AD but also for assessing the effectiveness of novel interventions (Horvath et al., 2018). Given that cognitive, perceptual, linguistic, emotional, and motor processes are fast, EEG and related methods can capture cognitive dynamics within tens to hundreds of milliseconds. Characteristics of waveforms, spectral power, and event-related oscillatory responses can be useful for differentiating the cognitive status, and the differences have shown a relationship with the scores of common neuropsychological tests assessing cognitive functions, which make EEG and related methods ideal for quantifying cognitive decline (Yener and Başar, 2013; Horvath et al., 2018). Many studies have used qEEG or ERP indices as biomarkers of MCI or early AD. Generally, patients with MCI or early AD have decreased alpha and beta band power and increased theta band power of EEG when compared with those of healthy aging adults (Jelic et al., 2000; Horvath et al., 2018). Furthermore, ERPs are useful for studying specific cognitive processes including sensory, motor, or perceptual processes as well as cognitive operations. Visually elicited ERPs and auditory ERPs, containing components such as N200 or P300, may be useful for identifying older adults who are at high risk of developing MCI and AD (Morrison et al., 2018, 2019). Among various metrics, a reduction and slowing of the P300 response is relatively consistent (Jiang et al., 2015). However, this phenomenon can also be observed in other types of dementia, for example, vascular dementia (Egerházi et al., 2008).

Impairment in episodic memory is commonly seen in patients with MCI-AD, reflecting a declining ability to learn and remember new information. In this regard, more attention should be paid to ERP components related to long-term memory, with a specific focus on episodic memory retrieval (Kappenman and Steven, 2011). ERPs are acquired and tested during separate test phases of a verbal or image recognition memory task (Dzulkifli et al., 2006; Waninger et al., 2018). Many studies have consistently reported retrieval-related effects, termed as “old/new effects,” that take the form of different ERPs for old (studied/previously seen) test items relative to new (non-studied/not seen) items. The ERPs show two effects. One effect is the early mid-frontal difference that occurs in the range of 300–500 ms after onset, which was first called “FN400” by Curran (1999). According to the dual-process theory (Mickes et al., 2009), the FN400 effect is related to familiarity, a sense reflecting the assessment of the experimental familiarity of a test item (Curran and Hancock, 2007). Some studies have hypothesized that this effect is related to an implicit form of memory called “conceptual fluency,” which is the subjective experience of ease or difficulty when processing information (Voss and Paller, 2006; Oppenheimer, 2008; Lucas et al., 2012). The other effect is the late positive potential (LPP) that is evoked in the range of 400–800 ms with a left parietal topography (Curran, 2000). The LPP is linked to the action of retrieving specific information.

Recent studies have shown that individuals with MCI have a reduction in the old/new effects that are generally observed in healthy control (HC) subjects (Yang et al., 2015; Waninger et al., 2018) and the reduction were consistent with neuropsychological assessments. Based on the above information, ERPs could be a suitable alternative owing to their sensitivity, low cost, and accessibility during the prescreening procedure to identify individuals with MCI.

In the present study, we performed an image recognition memory task together with response time measures and compared the outcomes between a cohort of patients diagnosed with MCI-AD and age-matched HC subjects. We adopted stricter inclusion criteria for the MCI-AD cohort by combining core clinical standards and CSF biomarkers, thus extending the results to early stage AD more precisely. Additionally, given that pictures with different emotional messages influence old/new responses (Weiss et al., 2008), we used neutral images collected from the International Affective Picture System (IAPS) to avoid any bias. The present study aimed to discover potential features during the retrieval of episodic memory to validate the ERP tasks as sensitive biomarkers for early stage AD. We hypothesized that patients with MCI-AD will have suppressed old/new effects in the image recognition memory task and that the ERP measurements may correlate with cognitive function evaluation.

## 2. Materials and methods

### 2.1. Participants

A total of 24 patients with MCI-AD and 31 HC subjects were enrolled in this study from the Department of Neurology at Nanjing Brain Hospital between October 2020 and May 2022. A diagnosis of MCI-AD was made only when core clinical criteria and a positive biomarker reflecting Aβ deposition, as proposed by the National Institute on Aging and Alzheimer’s Association (NIA-AA) Working Group, were satisfied simultaneously (Albert et al., 2011).

The inclusion criteria of patients were as follows: right-handed adults aged 50–79 years old; no obvious visual or hearing impairment; at least primary school education (6 years of education); chief complaint of memory impairment and objective evidence of impairment, with Mini-Mental State Examination (MMSE) scores being greater than 20; preserved independent ability in activities of daily living; and disease meeting the definition for Alzheimer’s continuum in accordance with the 2018 NIA-AA research framework (Jack et al., 2018). Subjects with any other systemic or brain diseases due to vascular, traumatic, and medical causes, such as hypothyroidism, cerebrovascular disease and brain trauma, that may have contributed to the cognitive decline were not included.

As control subjects, healthy elderly adults who were relatives of patients or community volunteers and had no evidence of a dementing or other neuropsychological disorder were recruited. The present study received approval from the Medical Research Ethics Committee of the Brain Hospital Affiliated to Nanjing Medical University. All participants enrolled in this study signed the informed consent before study initiation.

### 2.2. Clinical and neuropsychological assessments

The participants in both cohorts underwent comprehensive and standard clinical and neuropsychological assessments. Neuropsychological tests included the MMSE (Tombaugh and Mcintyre, 1992), Montreal Cognitive Assessment (MoCA) (Horton et al., 2015), Hasegawa’s Dementia Scale (HDS) (Gao, 1991), Clinical Dementia Rating (CDR) (Morris, 1993), Auditory Verbal Learning Test (AVLT) (Vakil and Blachstein, 1993), Hamilton Anxiety Scale (HAMA), and Hamilton Depression Scale (HAMD) (Bagby et al., 2004). Using these neuropsychological scales, experienced neuropsychologists evaluated participants’ general cognitive function, episodic memory, and emotional state.

### 2.3. Cerebrospinal fluid biomarkers

All 24 patients underwent a lumbar puncture to confirm the CSF profile of AD pathology including Aβ1-42 and Aβ40 concentrations, Aβ42/Aβ40 ratio, and phosphorylated tau and total tau levels. These biomarkers were detected using INNO-BIA AlzBio3 immunoassay kit-based reagents (Innotest, Fujirebio, Ghent, Belgium). The cutoff values were set based on the patient groups studied and laboratory experience summarized by Mulder et al. (2010).

### 2.4. Design of the image recognition task

The image recognition task involving a study block and a test block was designed to evaluate the memory encoding and retrieval processes. All 40 images, which could be seen frequently in daily life and had no positive or negative emotional bias, were selected carefully from the IAPS.

In the study block, the participants were presented with eight different pictures on the screen for maximum 1.0 s, with a 1.5 s interstimulus interval. These images were shown five times randomly on the left or right side of the cross-point “+,” and the participants were instructed to memorize each image to the best of their ability. The participants were then required to judge the position (right/left) of the pictures by pressing a button using their thumb (right/left thumb, respectively). When the participants entered the response within 1.0 s, the picture would disappear directly.

**FIGURE 1:**
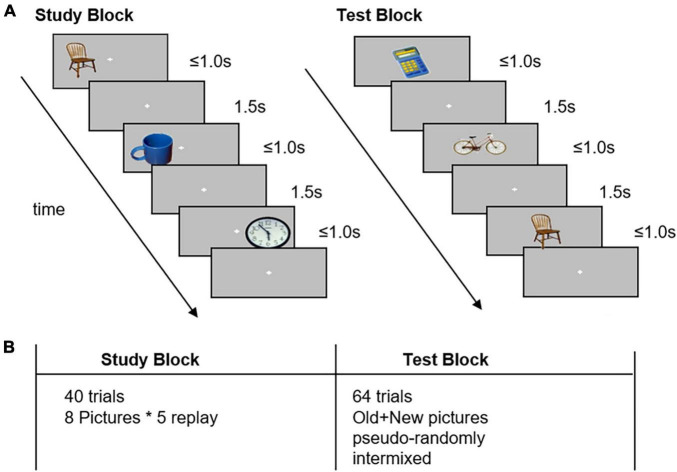
Task design in the image recognition memory task. Panel **(A)**: The experiment comprised a study block and a test block. In the study block, participants memorized the pictures and identified the relative location of the pictures to the fixation cross. In the test block, the participants were asked to identify previously seen items and press the button with their left thumb; if they were unable to recognize, they pressed the button with their right thumb. Panel **(B)**: The study consisted of 40 trials of neutral pictures. The test block consisted of 64 trials of studied (old) and non-studied (new) pictures, which were pseudo-randomly interleaved.

In the test block, a total of 64 trials comprising old pictures and 32 new pictures were shown on the center of the screen pseudo-randomly. Next, the participants were asked to judge whether they had already seen the picture in the study block. When the participants thought that they had seen the picture previously, they were asked to press the left button; otherwise, they were asked to press the right button. Even if the participants were unable to remember, they were asked to make a choice as soon as possible. Stimuli were generated and controlled using MATLAB and the Psychophysics Toolbox (Brainard, 1997). Example images are shown in Figure 1.

### 2.5. Behavioral data derived from the cognitive task

During the cognitive task, the participants were asked to finish the “right/left” or “old/new” judgment test. Accuracy was calculated as the percentage of correct trials per condition, including correct responses to old pictures (“Hits”) and correct rejections. Response time (RT) was defined as the time taken to press the button relative to the onset of trials, and the values were averaged separately for each condition, in a similar manner. Trials with incorrect responses or RT of < 200 or > 2,500 ms were excluded in response time analysis (Kappenman et al., 2014).

### 2.6. EEG recordings and preprocessing

The EEG signals were recorded at a sampling rate of 1,000 Hz using 64 electrodes in a BrainCap and amplified using BrainAmp DC (actiCHamp Plus, Brain Products GmbH, Gilching, Germany). The signal was low-pass filtered (100 Hz) online with a common reference between the Cz and Fz electrodes. Vertical electrooculography (EOG) was recorded from below the left eye, and horizontal EOG was recorded from the external right ocular canthus to detect blinks and eye movements. Electrode impedances were maintained below 10 kΩ. The recordings were preprocessed and analyzed offline using MATLAB (MathWorks Inc., Natick, MA, USA), EEGLAB software (MathWorks Inc., Natick, MA, USA), and FieldTrip toolbox. First, EOG channels were excluded, and then, raw EEG signals were re-referenced offline to global cerebral average reference, after which they were band-pass filtered offline at 0.1–40 Hz and down-sampled to 250 Hz. For continuous EEG data, independent component analysis was performed using EEGLAB software. EEG artifacts (e.g., blinks and eye movements as well as electrical and sensor noise) were isolated and removed using BESA Research automatic artifact correction (Berg and Scherg, 1994).

### 2.7. Analysis of event-related potentials (ERPs)

The continuous EEG data were epoched from 0.5 s before until 1.5 s after stimulus onset. Baseline was removed using data from 500 ms before stimulus onset. Trials with gross artifacts, for example, large physical movement artifacts, were excluded by visual inspection. The remaining trials in the recognition condition were used for ERP and time-frequency analyses. For each participant and each electrode, grand average ERP from cross trials of two event types was calculated and compared between groups and old/new conditions. Furthermore, we constructed difference waveforms by subtracting the ERP of new stimuli from that of old stimuli to isolate specific ERP components.

### 2.8. Time-frequency analysis

Time-frequency analysis using continuous wavelet transform was performed using FieldTrip toolbox. For each group and condition, power was computed by averaging across trials at each electrode location using a Morlet wavelet (width = 7) for a conservatively large frequency range of 4–20 Hz, with an analysis window being centered from −0.2 to 1.5 s, sliding in steps of 0.01 s. Time-frequency data were log_10_ transformed to normalize frequency distribution.

### 2.9. Statistical analysis

We used chi-square tests or independent-samples *t*-tests to evaluate group differences in gender distribution, age, education level, and cognitive function after testing normality and variance consistency. Data analysis was performed using IBM SPSS 26.0 software (SPSS Inc., Chicago, IL, USA). A repeated-measures analysis of variance (ANOVA) corrected using the Greenhouse–Geisser method and Scheirer-Ray-Hare test were applied to assess behavioral performance between different groups and conditions. Average amplitude values of difference waveforms across all electrodes, which were calculated from all groups, were calculated using independent-samples two-tailed *t*-test with the non-parametric cluster-based Monte Carlo permutation test (1,000 repetitions) to correct for multiple comparisons. Similarly, we compared power across all electrodes between groups using the cluster-based Monte Carlo permutation test (5,000 repetitions). Overall, statistical significance was assessed at a threshold of *p* = 0.025 at each tail.

We used Spearman’s Rho correlations to examine the relationship between behavioral or neural measures with neuropsychological performance. Finally, a receiver operator characteristic curve (ROC) was calculated to quantify the discriminatory power of each significantly altered biomarker between MCI-AD and HC groups. Multivariate binary logistic regression analysis was then conducted by combining the risk factors identified above. The predicted value of the model was calculated, and its discriminatory power was quantified by using ROC. The calibration was tested with the Hosmer–Lemeshow test for goodness of fit. *P* < 0.05 was considered statistically significant.

## 3. Results

### 3.1. Clinical characteristics

Table 1 lists the demographics and neuropsychological assessments for the MCI-AD and HC cohorts, wherein data are presented as mean values (± standard deviation). No significant differences were observed between the MCI-AD and HC groups regarding gender, education level, and age. The scores of general cognitive functions (MMSE, MoCA, and HDS scores) and episodic memory (AVLT: delayed recall and recognition) were lower in the MCI-AD group than in the HC group (*p* < 0.05). Overall, although all participants in the MCI-AD group were non-demented with a CDR score of less than 1, the MCI-AD group had higher activities of daily living and CDR scores than the HC group (*p* < 0.05). Moreover, patients who had evident anxiety and depression were excluded, but their HAMA and HAMD scores still tended to be higher than those of HC subjects (*p* < 0.05). All patients had positive results for CSF biomarkers, together with a decreased Aβ42 level or a reduced Aβ42/Aβ40 ratio. Some participants also had an elevated tau protein concentration.

**TABLE 1:** Demographics of subjects and neuropsychological test scores.

Characteristics	MCI-AD (Mean ± SD)	HC (Mean ± SD)	Test statistic	*P*-value
Sample size (male)	24 (13)	31 (14)	χ^2^ = 0.439	0.508
Age (years)	64.75 ± 9.377	65.06 ± 6.899	*T* = 0.143	0.887
Education (years)	12.25 ± 3.529	12.94 ± 3.326	*T* = 0.738	0.464
MMSE score	25.50 ± 2.571	28.06 ± 1.590	*T* = 4.292	<0.001
MOCA score	20.25 ± 3.859	26.35 ± 2.058	*T* = 7.016	<0.001
HDS score	27.44 ± 3.146	30.63 ± 1.565	*T* = 4.553	<0.001
AVLT delayed recall	1.58 ± 2.339	5.29 ± 2.479	*T* = 5.635	<0.001
AVLT recognition	16.29 ± 6.464	22.29 ± 1.637	*T* = 4.438	<0.001
ADL	21.38 ± 1.789	20.03 ± 0.180	*T* = −3.662	<0.001
HAMA	5.00 ± 2.964	2.84 ± 2.099	*T* = −3.032	0.004
HAMD	3.67 ± 2.792	2.00 ± 2.113	*T* = −2.521	0.015
**CSF biomarkers**				
Aβ42↓(%)	23 (95.83)	–	–	–
Aβ42/Aβ40 ratio ↓(%)	15 (62.50)	–	–	–
t-Tau↑(%)	14 (58.33)	–	–	–
p-Tau↑(%)	12 (50.00)	–	–	–

MCI-AD, mild cognitive impairment due to Alzheimer’s disease; HC, healthy control; MMSE, Mini Mental State Examination; MoCA, Montreal Cognitive Assessment; ADL, activities of daily living; HDS, Hasegawa Dementia Scale; AVLT, Auditory Verbal Learning Test; CSF, cerebrospinal fluid; Aβ42, amyloid-β (1-42); Aβ40, amyloid-β (1-40); t-Tau, total tau; p-Tau, tau phosphorylated at threonine _181_.

### 3.2. Behavioral results

**FIGURE 2:**
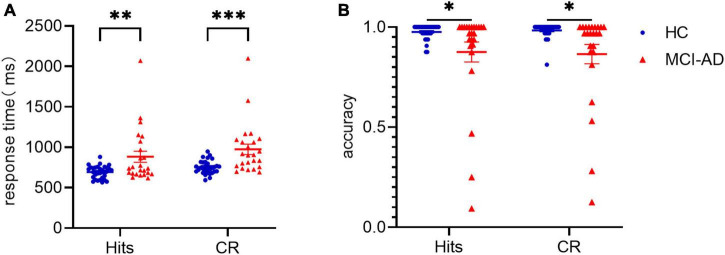
Data of behavioral performance in the HC and MCI-AD groups. Panel **(A)**: Average response time to old pictures (“Hits”) and new pictures (correct rejection) between the two groups. Panel **(B)**: Average accuracy over the two conditions and two groups. * indicates *p* < 0.05. ** indicates *p* < 0.01. *** indicates *p* < 0.0001. Error bars represent standard error of the mean.

To assess participants’ behavioral performance during the task, we performed a 2 × 2 repeated-measures ANOVA with factors of condition (old or new pictures) and group (MCI-AD or HC group). The results presented in Figure 2 showed significant differences in the main effects of conditions [*F*(1,53) = 17.610, *p* < 0.001, $\eta_{p}^{2}$ = 0.249] and groups [*F*(1,53) = 12.691, *p* = 0.001, $\eta_{p}^{2}$ = 0.03], but no significant differences in the interaction of condition × group [*F*(1,53) = 0.809, *p* = 0.372, $\eta_{p}^{2}$ = 0.015], driven by a shorter RT in the old picture condition and the HC group. Given that the accuracy data didn’t meet the assumptions of normality and homogeneity of variance, we performed Scheirer-Ray-Hare test. We also found significant differences in the main effects of group (*H* = 9.3612, *p* = 0.002) but not in the effects of condition (*H* = 0.1448, *p* = 0.704) or interaction (*H* = 0.8839, *p* = 0.347) when we set accuracy as the dependent variable, indicating that accuracy was mainly affected by cognitive state, not by event type. $\eta_{p}^{2}$ indicates the partial eta-squared, a measure of effect size used in ANOVA.

### 3.3. ERP analysis results

**FIGURE 3:**
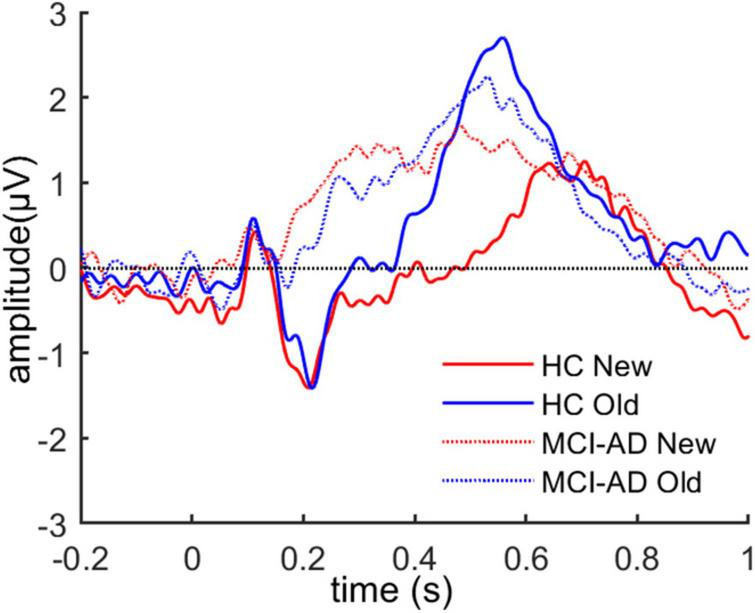
The grand average ERP waveforms over left parietal electrodes for both old and new images across the two groups.

We assessed whether old and new pictures elicited different patterns of brain activity in the all participants. For this assessment, the grand average ERP time-locked to stimulus onset of the IAPS images over left parietal electrodes (CP1, CP3, P1, and P3) was calculated for both conditions, which are presented in Figure 3. The LPP around 550 ms over the left parietal region could be seen in both groups.

**FIGURE 4:**
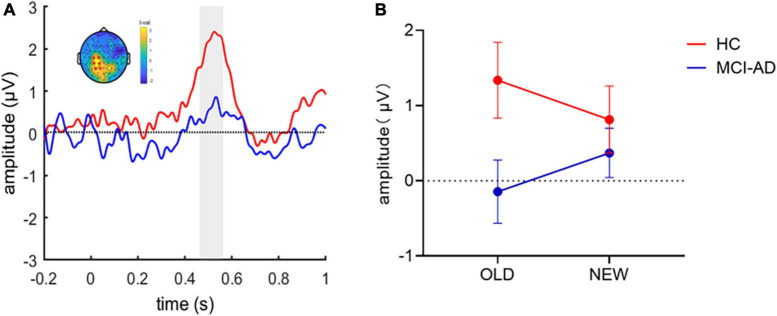
ERP old/new effect in the image recognition memory task. Panel **(A)**: Old-new difference waveforms between the two groups. A cluster-based permutation analysis between the two conditions revealed that the HC group had greater old/new difference waveform amplitude than the MCI-AD group over the left parietal regions. The shadow indicates the temporal window of significance (*p* < 0.05, corrected). Panel **(B)**: Average amplitude of ERPs at a present electrode (i.e., P3) for each group and condition. (Error bars represent standard error of the mean).

Then, we constructed difference waveforms to compare the difference in the old/new effects (old-new) between the two groups (Figure 4A). The difference waveforms between groups were analyzed using a cluster-based Monte Carlo permutation test. The analysis revealed a significant cluster that showed that a higher positive amplitude of difference waveforms in the HC group, which lasted for 470–570 ms after stimulus onset and occurred at the left parietal electrodes on the scalp. To assess the variation in the old or new ERP effects between groups, we calculated the average ERP activity at a represent electrode (i.e., P3) and performed repeated-measures ANOVA on condition (old and new images) and group (HC and MCI-AD groups; Figure 4B). However, the main effects of group [*F*(1,53) = 2.778, *p* = 0.101, $\eta_{p}^{2}$ = 0.050] and condition [*F*(1,53) < 0.001, *p* = 0.989, $\eta_{p}^{2}$ < 0.001] were not marked, and the interaction effect [*F*(1,53) = 3.889, *p* = 0.054, $\eta_{p}^{2}$ = 0.068] was marginally significant. Furthermore, the simple effect analysis indicated that the simple effect of group was sharply different in the “old image” condition [*F*(1,53) = 4.714, *p* = 0.034, $\eta_{p}^{2}$ = 0.082]. No significant simple effect of condition was observed.

### 3.4. Time-frequency analysis results

**FIGURE 5:**
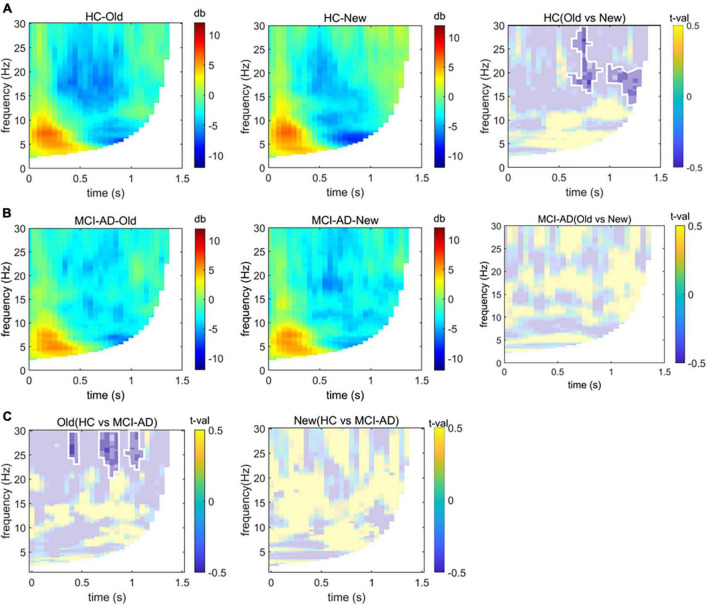
Time-frequency analysis. Panels **(A,B)**: Group-based average changes in spectral power over the left parietal electrodes induced by old or new images in the HC and MCI-AD groups. The difference between old image and new image conditions is also displayed based on the significant cluster (*p* < 0.05), marked with opaque color patches and circled by white lines. Panel **(C)**: Differences in spectral power over time elicited by old or new image stimuli between groups (*p* < 0.05, non-parametric cluster-based Monte Carlo permutation test).

We found a significant group-by-condition interaction effect [*F*(1,54) = 115.02, *p* < 0.001], indicating the effect of condition differs between groups. The cluster-based non-parametric test revealed differences in the neural oscillatory response to old or new images in the HC cohort along the temporal × frequency dimensions over the left parietal electrodes. The results revealed a significant cluster initiating at 600 ms from stimulus onset, comprising relatively high-frequency activity in the beta band (13–30 Hz; Figure 5A). No significant cluster was found in the MCI-AD cohort between conditions (Figure 5B). We could not analyze the spectrum over low frequency such as the delta and theta bands precisely because the trial length was too short (<1 s). We could merely observe the old responses accompanied by a decrease in beta power, and this effect was seen over the left parietal region of the scalp, limited to the HC cohort. Furthermore, we tried to accumulate more information about the decrease in spectral power over time elicited by old or new stimuli between the groups. The results showed that the spectral power changes over time were different between the groups in the old image condition but not in the new image condition (threshold of *p* = 0.05; Figure 5C). To make up for the lost information of the delta and theta bands, we simply extracted data of relative power through the Morlet wavelet power spectrum analysis. The relative power of the delta band showed noticeable difference between the groups (*p* = 0.004), while no significant difference was found in the theta band (*p* = 0.10).

### 3.5. Correlation of behavioral or neural measures with neuropsychological performance

**FIGURE 6:**
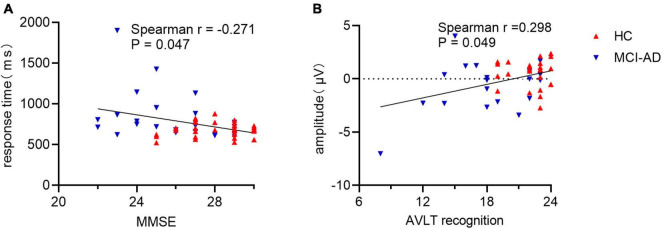
Correlation of behavioral response or old/new effect with cognitive assessment. Panel **(A)**: Correlation between response time and MMSE scores in all participants. Panel **(B)**: Correlation between mean amplitude of late old/new difference waveform and AVLT recognition scores. MMSE, Mini Mental State Examination; AVLT, Auditory Verbal Learning Test.

A significant and negative correlation was observed between behavioral performance and MMSE score (Spearman *r* = −0.271, *p* = 0.047; Figure 6A). Participants with a higher score tended to make a faster response to old images. The old/new effect measured as the average amplitude values of difference waveforms at the P3 electrode showed significant correlation with AVLT recognition scores (Spearman *r* = 0.298, *p* = 0.049; Figure 6B). A substantial correlation was not observed between behavioral performance and old/new effect measures or between MMSE scores and old/new effect measures (all *p* > 0.05).

### 3.6. Classifying patients with MCI-AD based on neural image and behavioral recognition memory performance

**FIGURE 7:**
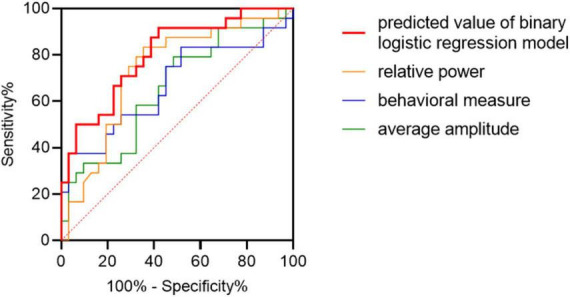
Classification of patients with MCI-AD from HC adults. Receiver operator characteristics (ROC) plots indicating the classification accuracy of MCI-AD versus HC using separate features and combined feature. Taking diagonal as the reference line, ROC curve above the line has classification significance.

Patients with MCI-AD could be discriminated from healthy elderly adults in the HC cohort using the predicted value of binary logistic regression model combing combining the characters of the average amplitude, relative power and behavioral measure (area under the receiver operating characteristic curve (AUC) = 0.80, 95% confidence interval (CI): 0.69–0.92, *p* < 0.001; Figure 7). Discrimination performance was moderate using single character such as the average amplitude (AUC = 0.66, 95% CI:0.51–0.80), the relative power (AUC = 0.73, 95% CI:0.60–0.87) and the behavioral measure (AUC = 0.67, 95% CI:0.52–0.82). The amplitude was measured by values of difference waveforms at the P3 electrode in the recognition condition. Relative power of the delta band providing frequency information was selected, and the behavioral measure was calculated by combining measures of processing speed and accuracy, namely, equal to the product of mean RT and accuracy divided by the sum of them.

## 4. Discussion

The current study examined the different behavioral and ERP measures in the image recognition memory task between the MCI-AD and HC cohorts. To summarize, the MCI-AD cohort had a significantly longer response time to the pictures and lower accuracy, regardless of the old or new pictures. All participants responded faster in the old picture condition. Meanwhile, the MCI-AD cohort had significant differences in the electrophysiological measures during the image recognition task. Differences in the LPP components between the old and new image conditions existed in the HC cohort but not in the MCI-AD cohort, predominantly over the left parietal regions. We observed no significant differences between responses to old and new pictures in both groups along the temporal × frequency × spatial (scalp sensors) dimensions.

The most striking difference between the MCI-AD and HC cohorts was observed when comparing the amplitude of the old–new difference waveforms during the test block. In the present study, the amplitude of difference waveform was significantly suppressed in the MCI-AD cohort, when compared with the HC cohort, over the left parietal regions around 470–570 ms after stimulus onset according to the statistical analysis results. Patients in the MCI-AD cohort had a smaller recollection effect, which could be the result of poor retrieval in discrimination between the studied and non-studied pictures. This finding is consistent with that of other studies showing a relationship between late parietal old/new effects (400–800 ms) (Curran, 2000) and the ability to recollect specific pictures (Curran and Cleary, 2003). However, we did not find any notable difference in the FN400 old/new effect between groups, although a negative waveform did exist in both groups at 300–500 ms after stimulus onset. The FN400 old/new effect appears to be related to familiarity reflecting the ability of the cognitive process to differentiate old (studied) from new (non-studied) pictures and its inability to differentiate similar from studied pictures. Given this situation, all participants tended to share a similar sense of familiarity to studied and non-studied pictures. This may be because all pictures we selected for the test are commonly seen in daily life, such as chair, bicycle, and so on. Yet, retrieval of real-world events also elicited a high proportion of familiarity (Nicolás et al., 2021). Consequently, despite losing the difference of the FN400 old/new effect between groups, the results influenced by pre-experimental fluency were compatible with those of previous studies generally acknowledged (Curran and Hancock, 2007; Leynes et al., 2017; Andrew Leynes and Upadhyay, 2022). The LPP old/new effects still showed significant variation, wherein the MCI-AD cohort performed poorly in terms of retrieving episodic memory related to accurate source judgments of episodic context (Addante et al., 2012), while the HC cohort performed better in identifying old pictures that appeared in the study block of the experiment. Importantly, although the cluster-based permutation test of difference waveforms between groups showed significant differences, the separate repeated-measures ANOVA results for ERPs specific to old and new images between groups revealed that none of them showed a significant main effect, but, instead, they showed a significant trend in the interaction effect of group × condition, indicating that we may need more subjects.

Unexpectedly, correct recognition of old pictures elicited a decrease in beta power but not in theta or alpha power over the left parietal electrodes in the HC cohort, but this effect disappeared in the MCI-AD cohort. EEG theta oscillations are usually associated with retrieval of contextual information (Guderian and Düzel, 2005; Herweg et al., 2016), and alpha rhythms may be related to the retrieval of memories (Martín-Buro et al., 2019) or subjects’ emotional states (Siqi-Liu et al., 2018). The task conducted in the current study was partly responsible for uncovering the theta oscillations because of the transient presentation of a single trial, while wavelet transform resulted in lower time resolution in the low-frequency band. Modulation of beta oscillations in human EEG was observed mainly during tasks that combined the cognition and execution processes, for example, the decision-making task (Zabielska-Mendyk et al., 2018), which is consistent with the current experimental design.

The MCI-AD cohort differed from the HC cohort in the performance evaluated using response time and accuracy during the image recognition task. However, it was difficult to distinguish whether the ability of memory or sustained attention was responsible for this outcome (Smid et al., 2006). As expected, response time to new pictures was slower in both groups, partly because of familiarity-based processing, but the accuracy did not differ between conditions. Accuracy showed group difference but not condition difference or interaction effect which might be related to ceiling effect and relatively few stimuli to some degree. However, it’s challenging to balance the need for a sufficient number of trials for the ERP analysis with the need to keep the total duration of the experiment manageable to prevent attentional decline. It is also worthwhile to mention that sometimes longer RTs could be associated with better memory if they are driven by recollection rather than familiarity. RT condition differences may scale overall with mean RT so that we will consider it in our future analyses. Grand average response time was related to the scores of general cognitive functions, showing a significant and negative correlation and demonstrating that subjects lost the ability to receive and process new information gradually as the cognitive impairment increased. Meanwhile, electrophysiology measurements showed a significant and positive correlation with AVLT scores but not with MMSE scores, RT, or accuracy. The finding emphasized that the late old/new effect was consistent with the neuropsychological performance, especially in the retrieval of episodic memory. Fortunately, we collected the testing results of CSF for patients with MCI-AD. However, amyloid deposition showed no significant correlation with cognitive assessment or electrophysiological measurements. Finally, receiver operating characteristic curve analysis showed that the late old/new effect did have a moderate potential to indicate cognitive impairment on an individual basis and serve as a biomarker for detecting MCI-AD. The current amount of data was not enough for model validation and the classification effect of EEG features itself was still unsatisfactory. We will keep on paying attention and further verify it in future research.

The current study had several limitations, which should be mentioned. First, differences in the merely old/new effects were compared between groups while other self-generated or other-generated sources such as visual and lingual ones and concentration remained unknown. Second, old images were presented multiple times during the test phase. This design could potentially allow participants to determine whether an item was old based on whether it was recently repeated, rather than whether they remembered it from the study phase. Considering of the cognitive state of our participants with cognitive impairment, the number of old images they can learn during the study phase is limited. In future studies, we will consider strategies to increase the number of trials and reduce the repetition of stimuli, while still accommodating the cognitive and attentional limitations of our participants. Additionally, the data used in the present study are not sufficient to set standards, and longitudinal follow-up of the HC and MCI-AD cohorts is meaningful to explore the association of the loss of old/new effect with conversion to AD-related dementia.

## 5. Conclusion

Collectively, these data suggest impaired recognition memory in patients with MCI-AD from the perspective of ERPs. This damage is manifested by the loss of late old/new effects, decreased accuracy, lower response time to stimuli, and abnormal late beta rhythm. Even though these changes show no significant correlation with Aβ or tau levels in CSF, they provide a method for the assessment of cognition in dementia and, to a certain extent, contribute to the development of early diagnosis tools.

## Data availability statement

The raw data supporting the conclusions of this article will be made available by the authors, without undue reservation.

## Ethics statement

The studies involving human participants were reviewed and approved by the Medical Research Ethics Committee of the Brain Hospital Affiliated to Nanjing Medical University. The patients/participants provided their written informed consent to participate in this study.

## Author contributions

YW was mainly responsible for the research design, data collection, data analysis, and manuscript writing of this study. XY was mainly responsible for research design, data analysis, review, and editing. BS was mainly responsible for data collection and document retrieval. YY and WM was mainly responsible for data collection. JS was mainly responsible for conceptualization, resources, and project administration. All authors contributed to the article and approved the submitted version.
